# A STEPPED-CARE SOCIAL AND MENTAL HEALTH ENGAGEMENT INTERVENTION FOR OLDER ADULT PROTECTIVE SERVICES CLIENTS

**DOI:** 10.1093/geroni/igae098.1067

**Published:** 2024-12-31

**Authors:** Jason Burnett, Sophia Wasik, Melba Hernandez-Tejada, Ron Acierno

**Affiliations:** The University of Texas Houston, Houston, Texas, United States; The University of Texas Houston, Houtson, Texas, United States; The University of Texas Health Science Center, Houston, Texas, United States; The University of Texas Health Science Center, Houston, Texas, United States

## Abstract

Adult Protective Services (APS) clients are commonly characterized as socially isolated and depressed, and recently, lonely. The only evidence-based interventions for this population target depression and none have focused on social isolation and loneliness (SIL). This study is the first to provide a formal assessment of loneliness in a large sample of APS clients and the first to test an intervention to address SIL in this population. Our study uses a randomized waitlist control design to test whether an intergenerational stepped-care social and mental health engagement approach reduces SIL and increases the likelihood of depression treatment for N = 200 older adult APS clients across Texas. We paired one pre-professional health student or criminal justice student (students) with each client to engage in 8 weekly friendly phone calls. The primary intervention targets are SIL and the secondary targets are depression treatment and reduction. At the end of each call students complete a brief mental health screen to assess for higher-level mental health treatment needs. If mental health needs were detected, participants were offered to be contacted by the UTHealth Trauma and Resilience Center. Prior to participant engagement, students received training on empathy-based communication, abuse, neglect, and exploitation and addressing objective or subjective indications of suicidal ideation. To date, we have enrolled 65 participants. In the intervention group, among those needing higher level mental health treatment, 73% agreed to being contacted. In this session we discuss preliminary findings on SIL and depression, and review intervention successes, challenges, solutions, and student perspectives.

